# Quantitative ^68^Ga-PSMA-11 PET and Clinical Outcomes in Metastatic Castration-resistant Prostate Cancer Following ^177^Lu-PSMA-617 (VISION Trial)

**DOI:** 10.1148/radiol.233460

**Published:** 2024-08-20

**Authors:** Phillip H. Kuo, Michael J. Morris, Jacob Hesterman, A. Tuba Kendi, Kambiz Rahbar, Xiao X. Wei, Bruno Fang, Nabil Adra, Rohan Garje, Jeff M. Michalski, Kim Chi, Johann de Bono, Karim Fizazi, Bernd Krause, Oliver Sartor, Scott T. Tagawa, Samson Ghebremariam, Marcia Brackman, Connie C. Wong, Ana M. Catafau, Taylor Benson, Andrew J. Armstrong, Ken Herrmann

**Affiliations:** From the University of Arizona, Tucson, Ariz (P.H.K.); Memorial Sloan-Kettering Cancer Center, New York, NY (M.J.M.); Invicro, Needham, Mass (J.H.); Mayo Clinic, Rochester, Minn (A.T.K., O.S.); Department of Nuclear Medicine, University Hospital Münster, Münster, Germany (K.R.); West German Cancer Center, Münster and Essen, Germany (K.R.); Dana-Farber Cancer Institute, Boston, Mass (X.X.W.); Astera Cancer Care, East Brunswick, NJ (B.F.); Indiana University Simon Comprehensive Cancer Center, Indianapolis, Ind (N.A.); Miami Cancer Institute, Baptist Health South Florida, Miami, Fla (R.G.); Washington University, St. Louis, Mo (J.M.M.); British Columbia Cancer Agency, Vancouver, British Columbia, Canada (K.C.); The Institute of Cancer Research and Royal Marsden Hospital, London, United Kingdom (J.d.B.); Gustave Roussy Institute, University of Paris-Saclay, Villejuif, France (K.F.); Rostock University Medical Center, Rostock, Germany (B.K.); Weill Cornell Medicine, New York, NY (S.T.T.); Novartis Pharmaceuticals, East Hanover, NJ (S.G.); Novartis Pharmaceuticals, Indianapolis, Ind (M.B.); Novartis Pharmaceuticals, Cambridge, Mass (C.C.W.); Novartis Pharmaceuticals, Geneva, Switzerland (A.M.C.); Novartis Pharmaceuticals, St. George, Utah (T.B.); Duke Cancer Institute Center for Prostate and Urologic Cancers, Duke University, Durham, NC (A.J.A.); and University Hospital Essen and German Cancer Consortium, Hufelandstr. 55, 45147 Essen, Germany (K.H.).

## Abstract

**Background:**

Lutetium 177 [^177^Lu]Lu-PSMA-617 (^177^Lu-PSMA-617) is a prostate-specific membrane antigen (PSMA)–targeted radioligand therapy for metastatic castration-resistant prostate cancer (mCRPC). Quantitative PSMA PET/CT analysis could provide information on ^177^Lu-PSMA-617 treatment benefits.

**Purpose:**

To explore the association between quantitative baseline gallium 68 [^68^Ga]Ga-PSMA-11 (^68^Ga-PSMA-11) PET/CT parameters and treatment response and outcomes in the VISION trial.

**Materials and Methods:**

This was an exploratory secondary analysis of the VISION trial. Eligible participants were randomized (June 2018 to October 2019) in a 2:1 ratio to ^177^Lu-PSMA-617 therapy (7.4 GBq every 6 weeks for up to six cycles) plus standard of care (SOC) or to SOC only. Baseline ^68^Ga-PSMA-11 PET parameters, including the mean and maximum standardized uptake value (SUV_mean_ and SUV_max_), PSMA-positive tumor volume, and tumor load, were extracted from five anatomic regions and the whole body. Associations of quantitative PET parameters with radiographic progression-free survival (rPFS), overall survival (OS), objective response rate, and prostate-specific antigen response were investigated using univariable and multivariable analyses (with treatment as the only other covariate). Outcomes were assessed in subgroups based on SUV_mean_ quartiles.

**Results:**

Quantitative PET parameters were well balanced between study arms for the 826 participants included. The median whole-body tumor SUV_mean_ was 7.6 (IQR, 5.8–9.9). Whole-body tumor SUV_mean_ was the best predictor of ^177^Lu-PSMA-617 efficacy, with a hazard ratio (HR) range of 0.86–1.43 for all outcomes (all *P* < .001). A 1-unit whole-body tumor SUV_mean_ increase was associated with a 12% and 10% decrease in risk of an rPFS event and death, respectively. ^177^Lu-PSMA-617 plus SOC prolonged rPFS and OS in all SUV_mean_ quartiles versus SOC only, with no identifiable optimum among participants receiving ^177^Lu-PSMA-617. Higher baseline PSMA-positive tumor volume and tumor load were associated with worse rPFS (HR range, 1.44–1.53 [*P* < .05] and 1.02–1.03 [*P* < .001], respectively) and OS (HR range, 1.36–2.12 [*P* < .006] and 1.04 [*P* < .001], respectively).

**Conclusion:**

Baseline ^68^Ga-PSMA-11 PET/CT whole-body tumor SUV_mean_ was the best predictor of ^177^Lu-PSMA-617 efficacy in participants in the VISION trial. Improvements in rPFS and OS with ^177^Lu-PSMA-617 plus SOC were greater among participants with higher whole-body tumor SUV_mean_, with evidence for benefit at all SUV_mean_ levels.

ClinicalTrials.gov identifier: NCT03511664

© The Author(s) 2024. Published by the Radiological Society of North America under a CC BY 4.0 license.

*Supplemental material is available for this article.*

## Graphical abstract

**Figure:**
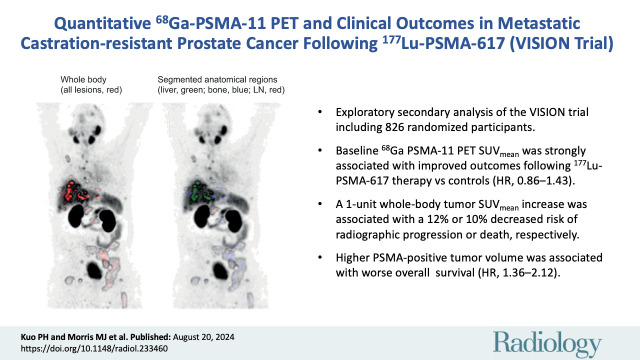

**Summary**

Visual ^68^Ga-PSMA-11 PET/CT eligibility criteria helped identify participants with metastatic castration-resistant prostate cancer who benefitted from ^177^Lu-PSMA-617 therapy in the VISION trial, with higher ^68^Ga-PSMA-11 uptake associated with improved outcomes.

**Key Results**

■ In this exploratory secondary analysis of the VISION trial that included 826 randomized participants, the baseline ^68^Ga-PSMA-11 PET mean standardized uptake value (SUV_mean_) was strongly associated with improved outcomes following ^177^Lu-PSMA-617 therapy versus controls (hazard ratio [HR] range, 0.86–1.43; *P* < .001).
■ A 1-unit whole-body tumor SUV_mean_ increase was associated with a 12% or 10% decrease in risk of radiographic progression-free survival or death, respectively.
■ Higher PSMA-positive tumor volume was associated with worse overall survival (HR range, 1.38–2.12; *P* ≤ .006).

## Introduction

Prostate-specific membrane antigen (PSMA) is a transmembrane glutamate carboxypeptidase that is highly expressed on the surface of prostate cancer cells (1,2). Lutetium 177 [^177^Lu]Lu-PSMA-617 (^177^Lu-PSMA-617) is a targeted radioligand therapy that delivers β-particle radiation selectively to PSMA-positive cells and the surrounding microenvironment. Although high PSMA expression in metastatic castration-resistant prostate cancer (mCRPC) has been associated with poor prognosis and reduced survival (3–5), it can also lead to improved uptake of ^177^Lu-PSMA-617 by target tumors and, therefore, potentially to a better treatment response. A prognostic nomogram developed from a multicenter retrospective study in patients with mCRPC demonstrated that higher PSMA expression by PET imaging was independently associated with better outcomes following ^177^Lu-PSMA-617 treatment (6). High PSMA-PET radiotracer uptake was also associated with improved response to ^177^Lu-PSMA-617 treatment versus cabazitaxel in the phase 2 TheraP trial (7).

In the phase 3 VISION trial, addition of ^177^Lu-PSMA-617 to protocol-permitted standard of care (SOC) prolonged radiographic progression-free survival (rPFS) and overall survival (OS) in participants with PSMA-positive mCRPC (8). Time to worsening in health-related quality of life and time to symptomatic skeletal events were also delayed (9). Baseline gallium 68[^68^Ga]Ga-PSMA-11 (^68^Ga-PSMA-11) PET/CT imaging was used to determine patient eligibility, based on visual assessment per central read rules (^68^Ga-PSMA-11 eligibility assessment criteria) designed specifically for VISION (8,10). A substudy required by the U.S. Food and Drug Administration showed that the VISION eligibility read rules used were readily learned and had good reproducibility (11).

Quantitative PSMA PET analysis could provide additional clinically relevant information on ^177^Lu-PSMA-617 treatment benefit and prognosis, given the correlation of ^68^Ga-PSMA-11 uptake with PSMA expression and thus with therapeutic targeting of PSMA in patients with mCRPC (12,13). Thus, the aim of this secondary analysis was to explore the association between quantitative baseline ^68^Ga-PSMA-11 PET parameters and treatment response and outcomes in the phase 3 VISION trial.

## Materials and Methods

### Study Design

This was a U.S. Food and Drug Administration–required, exploratory, secondary analysis of the phase 3 VISION trial of ^177^Lu-PSMA-617 in participants with mCRPC (ClinicalTrials.gov no. NCT03511664) (8). VISION was conducted in accordance with the principles of the Declaration of Helsinki and Good Clinical Practice guidelines. For sites located within the United States, Health Insurance Portability and Accountability Act forms were completed by the investigator and included all elements required by the U.S. Department of Health and Human Services Privacy Rule. All participants provided written informed consent. At each trial site, independent ethics review boards approved the trial protocol. The VISION trial and this exploratory analysis were funded by Novartis. All authors had control of the data and the information submitted for publication. The five authors who are employees of Novartis had full access to all data in the study and take responsibility for the integrity of the data and the accuracy of the data analysis.

### Participants

Complete inclusion and exclusion criteria have been published (8). Participants had pretreated PSMA-positive mCRPC, as assessed visually using ^68^Ga-PSMA-11 PET/CT per previously described central read rules (10,14). Participants had to have at least one PSMA-positive metastatic lesion and no exclusionary PSMA-negative lesions. Lesion PSMA positivity was defined as ^68^Ga-PSMA-11 uptake visually greater than that in the liver parenchyma, as determined by the sponsor’s central reader (8,10). Participants had progressive mCRPC and had previously received at least one androgen receptor pathway inhibitor and one or two taxane regimens (8). Participants with poor-quality PET images that were not analyzable were excluded from this secondary analysis.

### Intervention and Outcomes

Eligible participants were randomized 2:1 to ^177^Lu-PSMA-617 (7.4 GBq every 6 weeks for up to six cycles) plus protocol-permitted SOC or to protocol-permitted SOC only (8). Efficacy outcomes included in the present analysis were the two alternate primary end points (rPFS and OS), one of the key secondary end points (objective response rate [ORR]), and one of the additional secondary end points (prostate-specific antigen [PSA] response). End points were defined as previously described (8).

### Image Data Blinding

The image processing specialist was not blinded to essential clinical information (eg, injected dose and patient weight) needed for the imaging analysis. However, all institution-specific site and patient identifiers, patient demographics, clinical outcomes, and other clinical information not deemed essential for the quantitative analysis were masked.

### Quantitative Imaging Parameters

The mean standardized uptake value (SUV_mean_), maximum SUV (SUV_max_), PSMA-positive tumor volume, and tumor load were computed for each participant in the bone, liver, lymph node, soft tissue, and whole body (Appendix S1).

### Statistical Analysis

The present analyses were exploratory and noninferential and were not included in the study sample size calculation. Adjustment for multiple comparisons was not performed because the analyses were noninferential (all *P* values are nominal). Prespecified analyses were controlled for multiplicity and have been previously published (8). Statistical significance was assessed for each covariate based on a two-sided α = .05 significance level.

### Analysis Sets

All randomized participants were eligible for inclusion in this analysis. Each analysis set included all participants from the corresponding efficacy outcome analysis in VISION who had images of sufficient quality and had at least one PSMA-positive lesion in the anatomic region being analyzed (8). The OS analysis set included all randomized participants (8). The rPFS and PSA analysis sets included only participants randomized on or after March 5, 2019, as previously described (8). The ORR analysis set included all participants in the rPFS analysis set with disease evaluable by Response Evaluation Criteria in Solid Tumors version 1.1 at baseline (8).

### Univariable Analysis

Univariable Cox proportional-hazards models (15) were used to assess the individual predictive value of each quantitative ^68^Ga-PSMA-11 PET parameter in the whole body and in each anatomic region for rPFS and OS. Associations with ORR and PSA response were assessed using logistic regression (16). Analyses used a nominal two-sided α = .05.

### Multivariable Analysis

PET parameters found to be related to outcomes in the univariable analysis were included in a multivariable Cox proportional hazards model. Backward elimination was used to identify covariates that independently predicted rPFS, with a two-sided exclusion threshold of α = .05. Forward selection was then applied to all remaining covariates with a two-sided inclusion threshold of α = .05. Each covariate was then assessed in the final model. The same approach was applied to the other end points (OS, ORR, PSA response). Treatment group was also included as a covariate (^177^Lu-PSMA-617 plus SOC, SOC only). No clinical or other parameters were included as covariates.

### Quartile Analysis

Efficacy outcomes, rPFS and OS, were assessed in subgroups based on SUV_mean_ quartiles from the respective analysis set using the stratified Cox model to estimate hazard ratio (HR) and 95% CI, and the Kaplan-Meier method to estimate medians, percentiles, and 95% CI, as in the prespecified efficacy analyses (8). Statistical significance for HRs was evaluated as 95% CIs that excluded unity.

### Optimal Cut-Point Analyses

The optimal cut-point was defined as the value of SUV_mean_ that best separated participants on the basis of longer or shorter rPFS or OS benefit within a treatment arm. Threshold cut-point analyses used the linear predictor from the final multivariable model to provide the within-arm HR, 95% CI, and nominal *P* value (17). Additionally, optimal cut-points were explored using maximally selected rank statistics via the maxstat package of R (version 0.4.1; The R Foundation) with the surv_cutpoint function.

## Results

### Participant Characteristics

**Figure 1:**
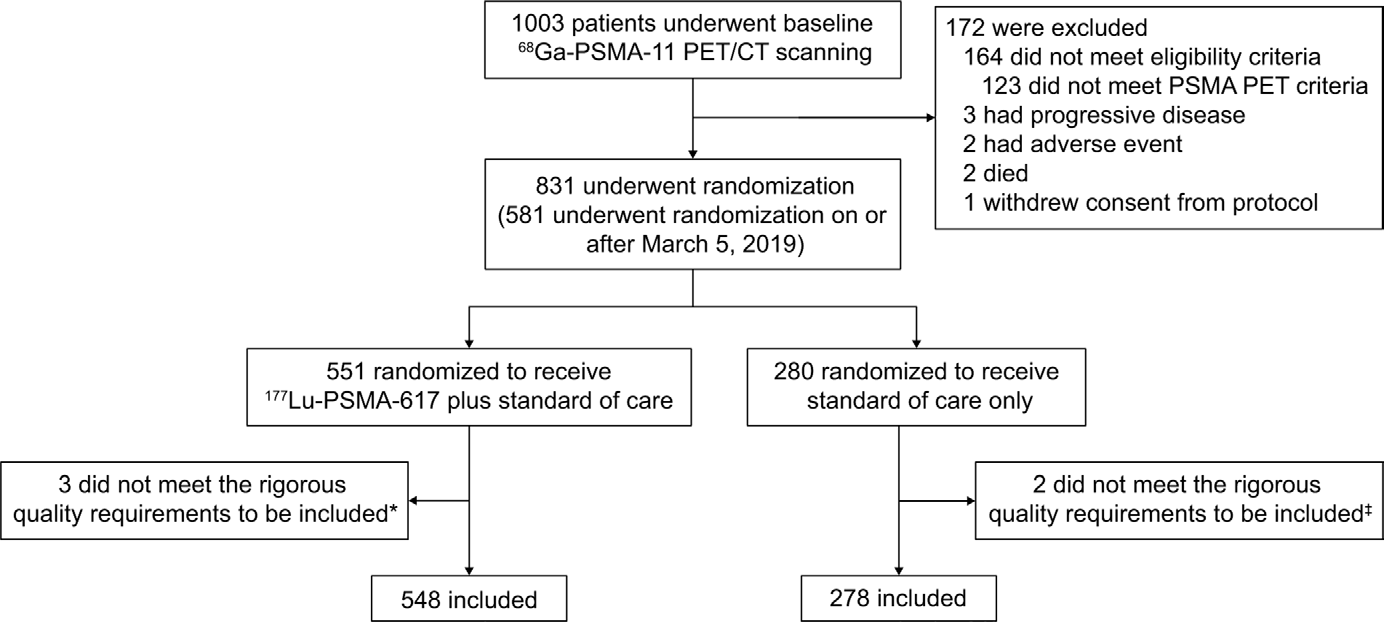
Flowchart for the VISION trial shows the randomization of participants and exclusion criteria for the participants included in the present analysis. * Reasons for exclusion included *(a)* separate fields of view, *(b)* incomplete field of view, and *(c)* imaging artifact that impeded quantification. ^‡ ^Reasons for exclusion included *(a)* use of units that could not be converted to standardized uptake value and *(b) *inadequate emission start time postinjection. PSMA = prostate-specific membrane antigen.

**Figure 2:**
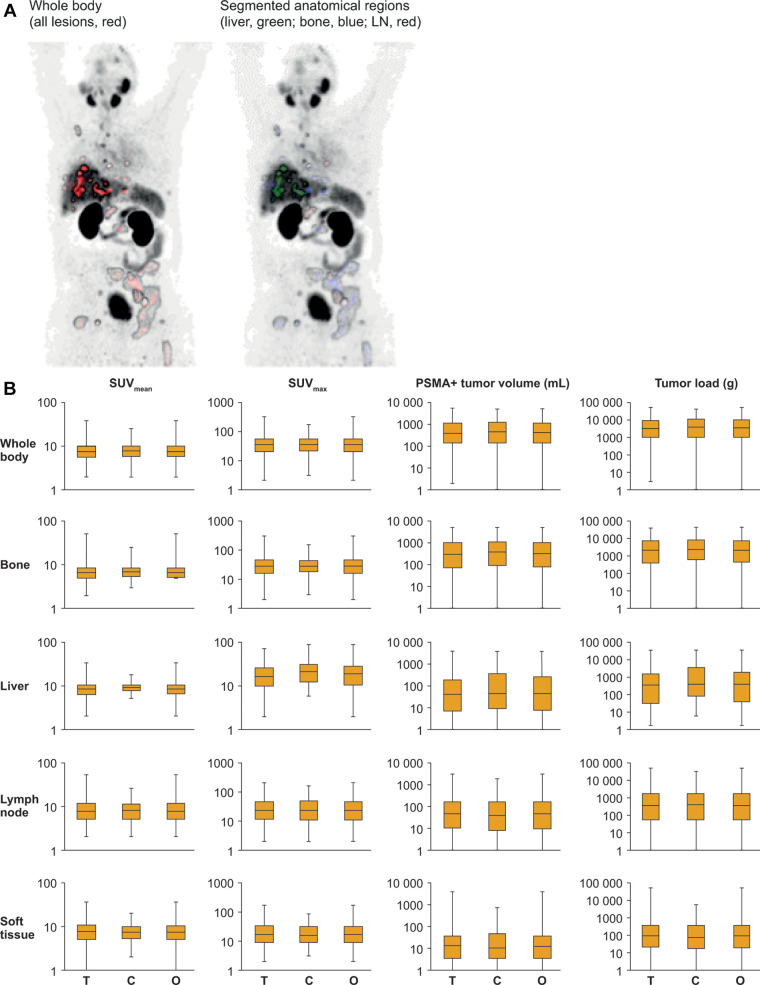
Segmentation of anatomic regions and distribution of quantitative ^68^Ga-PSMA-11 PET parameters. **(A)** Whole-body anterior coronal prostate-specific membrane antigen (PSMA) PET maximum intensity projection images in a 63-year-old White male participant with tumors in the liver, bone, and lymph node (LN) who had an initial prostate-specific antigen level of 181.9 ng/mL and Eastern Cooperative Oncology Group performance score of 0/1 show all PSMA-positive (PSMA+) disease as a single whole-body volume in red (left) and segmented according to anatomic region (right; bone lesions in blue, liver lesions in green, lymph node lesions in red). **(B)** Box plots show the distribution of quantitative ^68^Ga-PSMA-11 PET parameters for the study sample (*n* = 826) included in this analysis. Data are reported as medians and IQRs from the full analysis sets for bone (*n* = 761), liver (*n* = 109), lymph node (*n* = 559), soft tissue (*n* = 334), and whole body (*n* = 826) and stratified according to treatment (T; ^177^Lu-PSMA-617 plus standard of care [SOC]), control (C; SOC only), and overall (O; all participants). SUV_max_ = maximum standardized uptake value, SUV_mean_ = mean standardized uptake value.

Of 1003 participants who underwent baseline ^68^Ga-PSMA-11 PET/CT, 831 met all study eligibility criteria and were randomized (June 2018 to October 2019) in VISION (8). Of the 831 randomized participants, 826 had baseline ^68^Ga-PSMA-11 PET scans that met the quality requirements and were included in the present analysis (Table 1). The reasons for exclusion of five participants were as follows: incomplete field of view, separate fields of view, imaging artifact that impeded quantification, use of units that could not be converted to SUV, and inadequate emission start time postinjection. Of the 551 participants randomized to receive ^177^Lu-PSMA-617 plus SOC, 548 were included, and of the 280 randomized to receive SOC only, 278 were included in the present analysis (Fig 1). Quantitative parameters were successfully extracted for all included participants. Representative PET/CT images of whole-body and regional segmentation are shown in Figure 2A. The number of participants included in the analysis for each anatomic region and outcome is shown in Table 2.

**Table 1:**
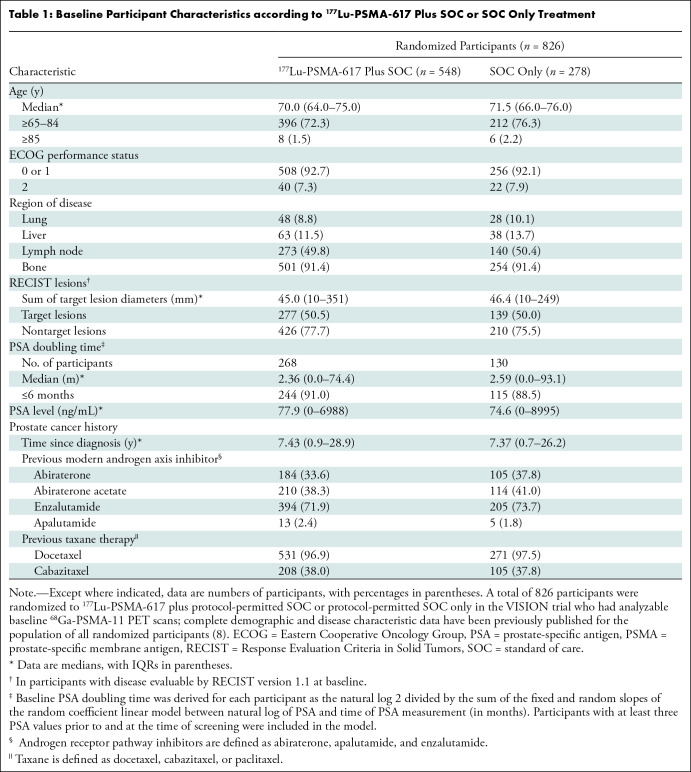
Baseline Participant Characteristics according to ^177^Lu-PSMA-617 Plus SOC or SOC Only Treatment

**Table 2:**
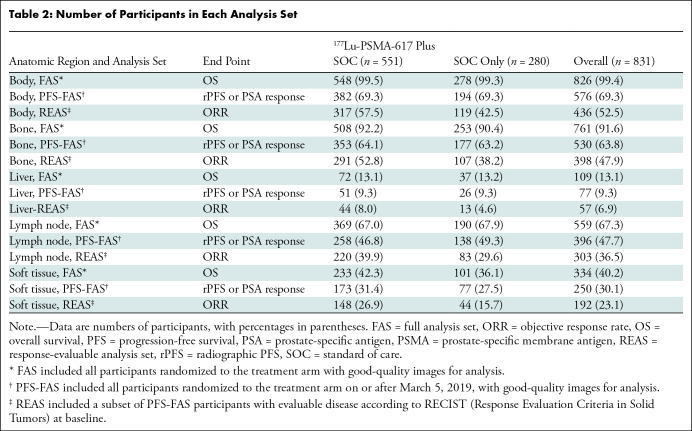
Number of Participants in Each Analysis Set

### Parameters and Associations

SUV_mean_, SUV_max_, PSMA-positive tumor volume, and tumor load were well balanced between the study treatment arms in the whole body and in bone, liver, lymph node, and soft tissue (Fig 2B). The median whole-body tumor SUV_mean_ was 7.5 (IQR, 5.7–9.9) in the ^177^Lu-PSMA-617 plus SOC arm, 7.7 (IQR, 5.8–10.0) in the SOC only arm, and 7.6 (IQR, 5.8–9.9) in the overall study sample (*n* = 826). Representative PET/CT images and data for participants with low and high SUV_mean_ are shown in Figure S1.

### Radiographic Progression-free Survival

**Figure 3:**
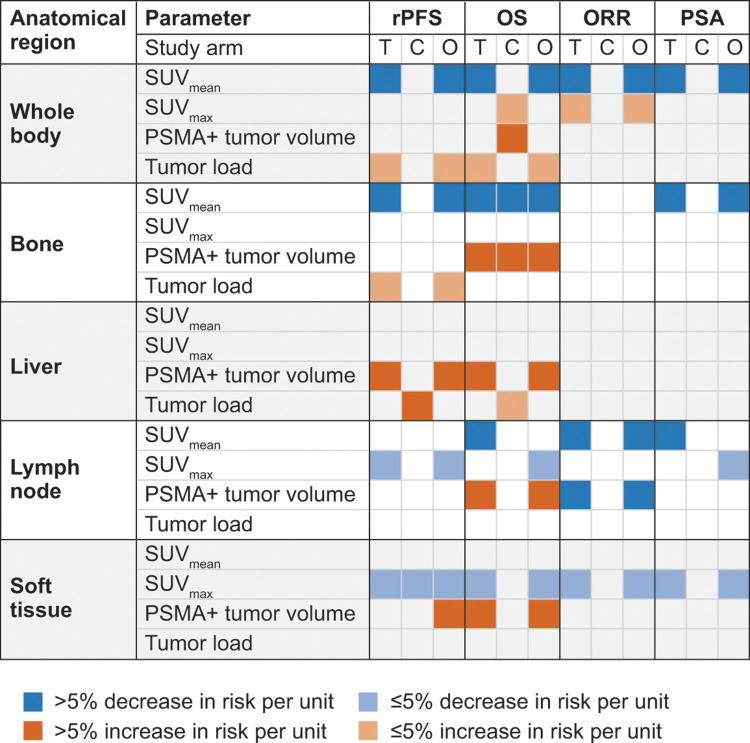
Association of quantitative ^68^Ga-PSMA-11 PET parameters with efficacy outcomes in the final multivariable model. In the chart, boxes show the level of association between each PET parameter per anatomic region and each clinical outcome. Blue and orange boxes represent statistically significant associations with improved and worse clinical outcomes, respectively (*P* < .05). White boxes indicate that no statistically significant associations were observed (*P* ≥ .05). Associations are shown within the ^177^Lu-PSMA-617 plus standard of care (SOC) treatment group (T), within the SOC only control group (C), or within the overall study sample (O), and the model was adjusted for study treatment (^177^Lu-PSMA-617 plus SOC or SOC only). The presence of prostate-specific membrane antigen–positive (PSMA+) tumors was included as a categorical variable but is not reported here for clarity. max = maximum, ORR = overall response rate, OS = overall survival, PSA = prostate-specific antigen, rPFS = radiographic progression-free survival, SUV_max_ = maximum standardized uptake value, SUV_mean_ = mean standardized uptake value.

Whole-body SUV_mean_ and tumor load were associated with rPFS (HR, 0.86 [95% CI: 0.82, 0.90; *P* < .001] and 1.02 [95% CI: 1.01, 1.04; *P* < .001], respectively) in the ^177^Lu-PSMA-617 plus SOC arm, with SUV_mean_ as the strongest predictor in the final treatment-adjusted multivariable model (Fig 3, Table 3). The multivariable model did not include SUV_mean_ in the SOC only arm. A 1-unit increase in whole-body tumor SUV_mean_ was associated with a 12% decrease in the risk of an rPFS event (radiographic progression or death) and a 1000-g increase in tumor load was associated with a 2% increase in the risk of an rPFS event in the overall study sample (*n* = 576).

**Table 3:**
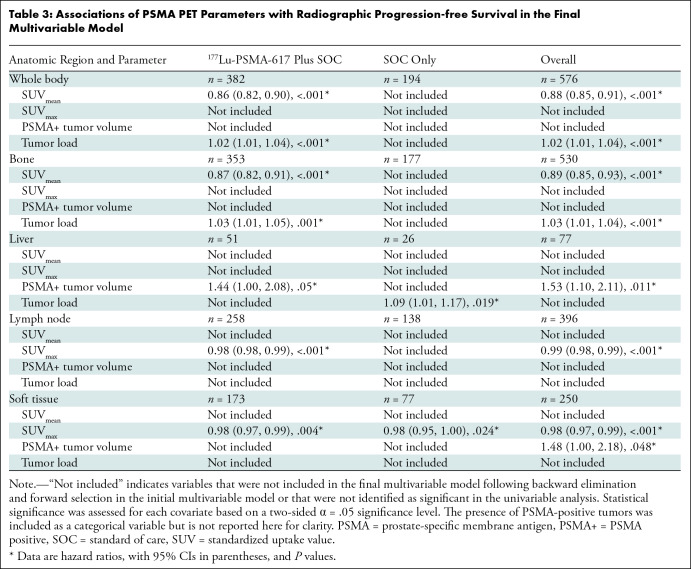
Associations of PSMA PET Parameters with Radiographic Progression-free Survival in the Final Multivariable Model

Regarding anatomic regions in the overall study sample, bone SUV_mean_ (HR, 0.89 [95% CI: 0.85, 0.93]; *P* < .001) and lymph node and soft-tissue SUV_max_ (HR, 0.99 [95% CI: 0.98, 0.99; *P* < .001] and 0.98 [95% CI: 0.97, 0.99; *P* < .001], respectively) were associated with improved rPFS. Bone tumor load (HR, 1.03 [95% CI: 1.01, 1.04]; *P* < .001) and liver and soft-tissue PSMA-positive tumor volume (HR, 1.53 [95% CI: 1.10, 2.11; *P* = .011] and 1.48 [95% CI: 1.00, 2.18; *P* = .048], respectively) were associated with reduced rPFS (Fig 3, Table 3).

**Figure 4:**
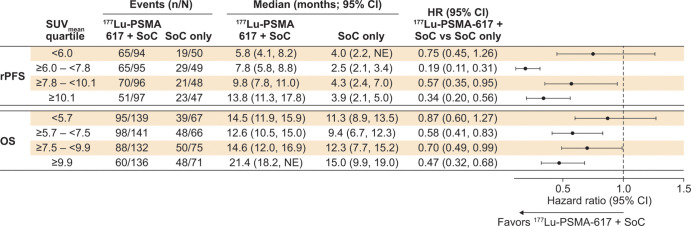
Chart shows median radiographic progression-free survival (rPFS) and overall survival (OS) according to whole-body tumor mean standardized uptake value (SUV_mean_) quartile, indicating statistically significant differences in the three upper quartiles for both rPFS and OS but not the lowest quartile. SUV_mean_ quartiles were derived from the SUV_mean_ of both study arms combined (^177^Lu-PSMA-617 plus standard of care [SOC] and SOC only). The statistical significance of the hazard ratios (HRs) for each quartile is indicated by 95% CIs that exclude unity. NE = not evaluable, PSMA = prostate-specific membrane antigen.

**Figure 5:**
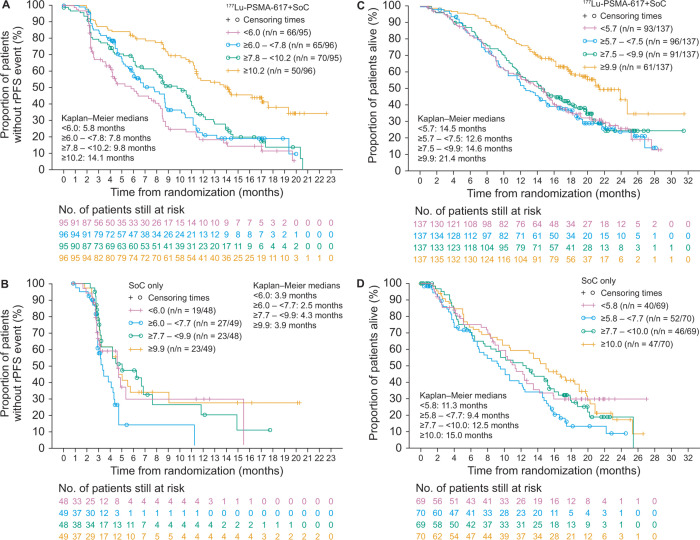
Kaplan-Meier curves show radiographic progression-free survival (rPFS) according to whole-body tumor mean standardized uptake value (SUV_mean_) quartile for **(A) **^177^Lu-PSMA-617 plus standard of care (SOC) (*n* = 382) and **(B)** SOC only (*n* = 194) treatment arms (body, progression-free survival, full analysis set). SUV_mean_ quartiles were derived from either the SUV_mean_ of the ^177^Lu-PSMA-617 plus SOC arm or the SOC only arm. PSMA = prostate-specific membrane antigen. Kaplan-Meier curves show overall survival according to whole-body tumor mean standardized uptake value (SUV_mean_) quartile for **(C) **^177^Lu-PSMA-617 plus standard of care (SOC) (*n* = 548) and **(D)** SOC only (*n* = 278) treatment arms (body, full analysis set). SUV_mean_ quartiles were derived from either the SUV_mean_ of the ^177^Lu-PSMA-617 plus SOC arm or the SOC only arm. PSMA = prostate-specific membrane antigen.

The median rPFS was longer in the ^177^Lu-PSMA-617 plus SOC arm versus the SOC only arm in all whole-body tumor SUV_mean_ quartiles, with the 95% CIs for HRs for the upper three quartiles excluding unity (Figs 4, 5A, 5B). In the highest SUV_mean_ quartile (≥10.1), median rPFS was 13.8 months in the ^177^Lu-PSMA-617 plus SOC arm and 3.9 months in the SOC only arm (HR, 0.34 [95% CI: 0.20, 0.56]). In the lowest SUV_mean_ quartile (<6.0), median rPFS was 5.8 months in the ^177^Lu-PSMA-617 plus SOC arm and 4.0 months in the SOC only arm (HR, 0.75 [95% CI: 0.45, 1.26]). In the ^177^Lu-PSMA-617 plus SOC arm, the median rPFS increased as the SUV_mean_ quartile increased (as judged by nonoverlap with the 95% CI of the neighboring estimate) (Fig 4).

### Overall Survival

Whole-body tumor SUV_mean_ and tumor load were associated with OS in the ^177^Lu-PSMA-617 plus SOC arm (HR, 0.88 [95% CI: 0.84, 0.91; *P* < .001] and 1.04 [95% CI: 1.03, 1.05; *P* < .001], respectively), with SUV_mean_ as the strongest predictor in the final treatment-adjusted multivariable model (Fig 3, Table 4). The multivariable model did not include SUV_mean_ in the SOC only arm. A 1-unit increase in whole-body tumor SUV_mean_ was associated with a 10% decrease in the risk of death, and a 1000-g increase in tumor load was associated with a 4% increase in the risk of death in the overall study sample (*n* = 576).

**Table 4:**
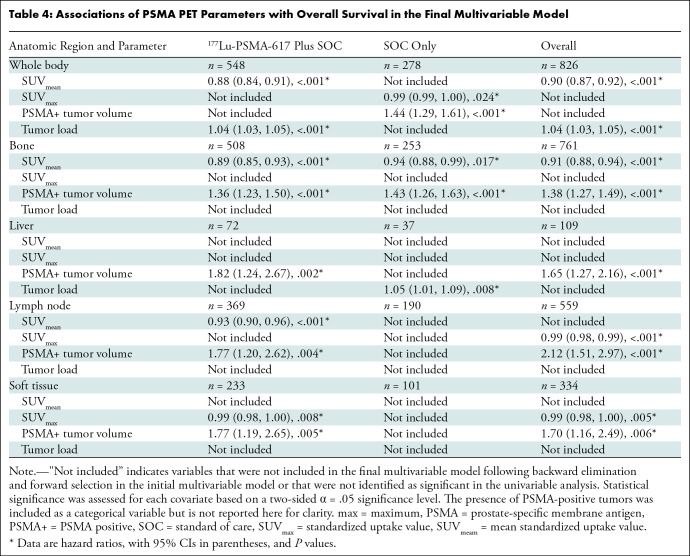
Associations of PSMA PET Parameters with Overall Survival in the Final Multivariable Model

Regarding anatomic regions in the overall study sample, bone SUV_mean_ (HR, 0.91 [95% CI: 0.88, 0.94]; *P* < .001), lymph node, and soft-tissue SUV_max_ (HR, 0.99 [95% CI: 0.98, 0.99; *P* < .001] and 0.99 [95% CI: 0.98, 1.00; *P* = .005], respectively) were associated with improved OS. Bone, liver, lymph node, and soft-tissue PSMA-positive tumor volume were associated with reduced OS (HR, 1.38 [95% CI: 1.27, 1.49; *P* < .001]; 1.65 [95% CI: 1.27, 2.16; *P* < .001]; 2.12 [95% CI: 1.51, 2.97; *P* < .001]; 1.70 [95% CI: 1.16, 2.49; *P* = .006], respectively) (Fig 3, Table 4).

The median OS was longer in the ^177^Lu-PSMA-617 plus SOC arm than in the SOC only arm in all whole-body tumor SUV_mean_ quartiles, with the 95% CIs for HRs for the upper three quartiles excluding unity (Figs 4, 5C, 5D). In the highest SUV_mean_ quartile (≥9.9), median OS was 21.4 months in the ^177^Lu-PSMA-617 plus SOC arm and 15.0 months in the SOC only arm (HR, 0.47 [95% CI: 0.32, 0. 68]). In the lowest SUV_mean_ quartile (<5.7), median OS was 14.5 months in the ^177^Lu-PSMA-617 plus SOC arm and 11.3 months in the SOC only arm (HR, 0.87 [95% CI: 0.60, 1.27]). In the ^177^Lu-PSMA-617 plus SOC arm, median OS was longer in the highest SUV_mean_ quartile compared with the lower three SUV_mean_ quartiles, in which median OS was similar (as judged by nonoverlap or overlap with the 95% CI of neighboring estimates).

### ORR and PSMA Response

Whole-body tumor SUV_mean_ was associated with improved ORR in the ^177^Lu-PSMA-617 plus SOC arm (odds ratio [OR], 1.39 [95% CI: 1.22, 1.58]; *P* < .001) (Fig 3, Table S1) in the final treatment-adjusted multivariable model. The multivariable model did not include SUV_mean_ in the SOC only arm. A 1-unit increase in whole-body tumor SUV_mean_ was associated with a 39% increase in the odds of objective response in the overall study sample (*n* = 436). SUV_max_ was associated with modestly reduced ORR in the ^177^Lu-PSMA-617 plus SOC arm (OR, 0.98 [95% CI: 0.96, 0.99]; *P* = .006). The multivariable model did not include SUV_max_ in the SOC only arm. Whole-body tumor SUV_mean_ was associated with improved odds of a confirmed PSA response in the ^177^Lu-PSMA-617 plus SOC arm (OR, 1.23 [95% CI: 1.16, 1.31]; *P* < .001) (Fig 3, Table S2) in the final treatment-adjusted multivariable model. A 1-unit increase in whole-body tumor SUV_mean_ was associated with a 23% increase in the odds of confirmed PSA response in the overall study sample (*n* = 576). ORR and PSA response according to SUV_mean_ quartile are shown in Table S3.

### SUV_mean_ Cut-Point Analyses

**Figure 6:**
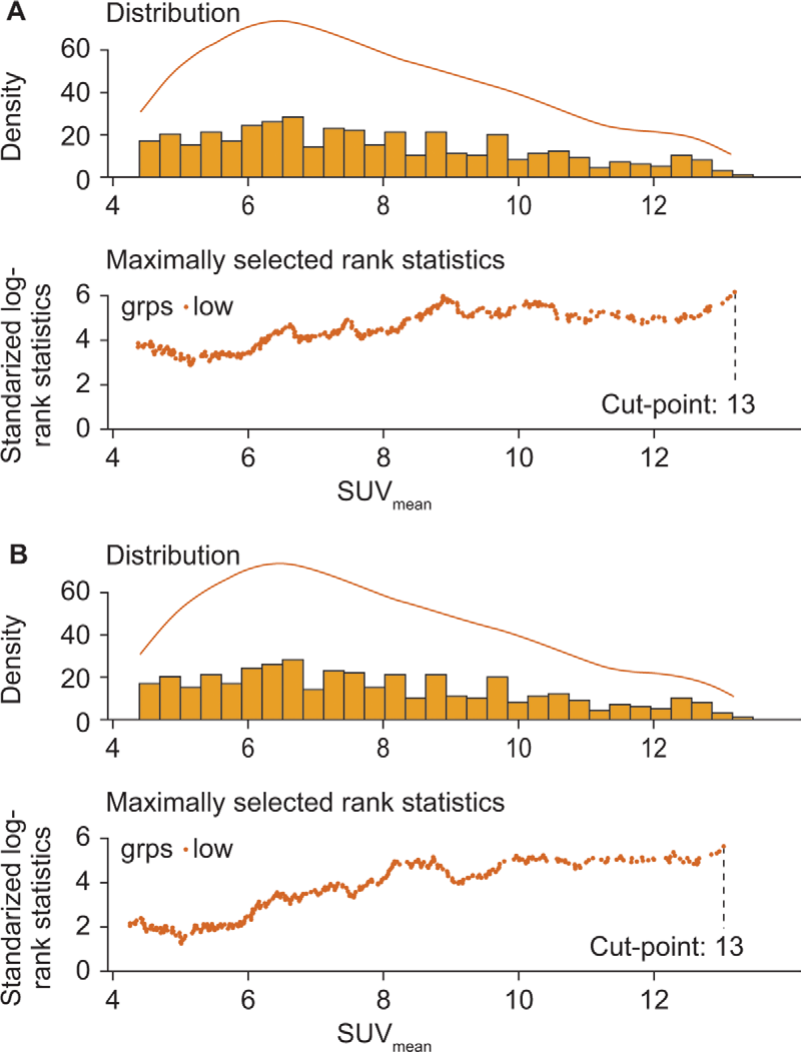
Maximally selected rank statistics analysis for identification of optimal whole-body tumor mean standardized uptake value (SUV_mean_) cut-points for **(A)** radiographic progression-free survival (rPFS) and **(B)** overall survival (OS) in the ^177^Lu- PSMA-617 plus standard of care arm (rPFS, *n* = 382; OS, *n* = 548). For both **A** and **B**, the top panel shows the SUV_mean_ histogram and the bottom panel shows the standardized log-rank statistics using different SUV_mean_ values as cutoffs. The standardized log-rank statistics demonstrate an upward trend, suggesting no ideal cutoffs can be identified within the range of SUV_mean_ values in the VISION trial. grps = groups, PSMA = prostate-specific membrane antigen.

Next, it was attempted to determine an optimal whole-body tumor SUV_mean_ threshold for separating participants receiving ^177^Lu-PSMA-617 into subgroups with longer or shorter rPFS or OS by analyzing all potential cut-points. No meaningful optimal cut-point within the ^177^Lu-PSMA-617 plus SOC arm for rPFS or OS was found using maximally selected rank statistics analysis (Fig 6). Analyses using the linear predictor from the final multivariable model also could not identify a meaningful optimal cut-point within the ^177^Lu-PSMA-617 plus SOC arm for rPFS and OS. SUV_mean_ cut-points between 5 and 20 for rPFS and 7 and 18 for OS were all associated with statistically significant within-arm HRs (*P* ≤ .012 and *P *≤ .005, respectively) (Tables S4, S5). All evaluated cut-points were similar in their ability to distinguish participants receiving ^177^Lu-PSMA-617 plus SOC on the basis of longer or shorter rPFS or OS, with no identifiable optimal threshold.

## Discussion

Lutetium 177 [^177^Lu]Lu-PSMA-617 (^177^Lu-PSMA-617) is a prostate-specific membrane antigen (PSMA)–targeted radioligand therapy for metastatic castration-resistant prostate cancer (mCRPC). This secondary, exploratory, quantitative analysis of the VISION trial explored the benefits of ^177^Lu-PSMA-617 in randomized participants with extensively pretreated PSMA-positive mCRPC. Treatment included either ^177^Lu-PSMA-617 (7.4 GBq every 6 weeks for up to six cycles) plus standard of care (SOC) or SOC only. We found that the whole-body tumor mean standardized uptake value (SUV_mean_) was the best predictor of ^177^Lu-PSMA-617 efficacy for all outcomes tested. A 1-unit whole-body tumor SUV_mean_ increase was associated with a 12% and 10% decrease in risk of a radiographic progression-free survival event (rPFS) and death, respectively. ^177^Lu-PSMA-617 plus SOC prolonged rPFS and overall survival (OS) in all SUV_mean_ quartiles compared with SOC only. Increased baseline tumor load was associated with worse rPFS and OS.

No optimal cut-point for SUV_mean_ was identifiable that could separate participants receiving ^177^Lu-PSMA-617 plus SOC into subgroups with longer or shorter rPFS and OS. The correlation of improved outcomes with higher whole-body SUV_mean_ values is linear, indicating that even participants with the lowest SUV_mean_ in the VISION population had the potential for improved rPFS and OS with ^177^Lu-PSMA-617. High SUV_mean_ values indicate high average PSMA expression levels, which may promote tumor binding and uptake of ^177^Lu-PSMA-617, leading to greater delivery of radiation and greater antitumor activity (18,19). In agreement with our present findings, high SUV_mean_ was associated with improved treatment response to ^177^Lu-PSMA-617 versus cabazitaxel in the phase 2 TheraP study (7,20).

Median OS appeared to be longer in the highest whole-body tumor SUV_mean_ quartile in the SOC only arm, suggesting that high average PSMA expression may be a favorable prognostic factor in mCRPC. In a small observational study (*n* = 16), patients who were ineligible for ^177^Lu-PSMA-617 owing to low PSMA expression or discordant fluorodeoxyglucose-positive PSMA-negative disease had a median OS of only 2.5 months (21). These findings contrast with previous reports that high PSMA expression is associated with poor outcomes in patients with prostate cancer and mCRPC (3,4,22). Therefore, the prognostic value of PSMA PET SUV_mean_ in prostate cancer merits further evaluation.

Increased baseline whole-body PSMA-positive tumor load and increased bone and liver tumor volume and tumor load were associated with decreased rPFS or OS. In VISION trial participants, these measures mainly reflect the extent and pattern of metastatic disease, rather than tumor PSMA expression, because patients with exclusionary PSMA-negative lesions were ineligible. Therefore, these findings are consistent with patients with more severe metastatic disease having less favorable clinical outcomes than those with less severe disease.

Visual assessment of ^68^Ga-PSMA-11 PET/CT scans according to the VISION trial read rules is an accurate and straightforward means of identifying candidates for ^177^Lu-PSMA-617 treatment (11). Visual PET interpretation is the most widely used method in clinical practice, and comparison of tumor uptake with liver uptake is simple. Using quantitative PET for patient selection is difficult in clinical practice because SUV measurements not obtained in a well-conducted clinical trial are subject to variability. This is because of technical factors such as the PET scanner used and the acquisition and reconstruction parameters (23–26). Semiautomatic segmentation of lesions into anatomic regions was used to extract quantitative PET parameters in the present study. Further research could help standardize whole-body tumor SUV_mean_ quantification using automated or artificial intelligence–enabled software, with normalization of uptake to comparator organs such as the liver or salivary glands. Therefore, SUV_mean_ may have a role in patient selection in the future.

Whether VISION-ineligible patients, as defined by visual PSMA PET/CT read rules, could benefit from ^177^Lu-PSMA-617 treatment remains untested (as for other PSMA PET selection criteria, such as those used in TheraP) (20,27). Future investigative directions include quantifying the tumor-absorbed dose and normal organ-absorbed dose, enhancing ^68^Ga-PSMA-11 PET sensitivity, and standardizing the reporting of PSMA PET/CT. The relationship between timing of SOC and PSMA PET also warrants investigation because hormonal therapy may influence expression of PSMA (28–30).

Our study had limitations. First, the VISION trial was not powered for analysis of efficacy in subgroups identified by quantitative PET. Second, quantitative PET was performed centrally, and study sites were required to follow standard protocols, but devices and reconstruction parameters may have differed among study sites. Third, results obtained with ^68^Ga-PSMA-11 are not applicable to other radiotracers; therefore, our methods and findings are not directly applicable to clinical practice. For this reason, we did not assess the reproducibility of contouring or the time taken for analysis. Finally, the VISION trial was not designed to compare ^177^Lu-PSMA-617 with cabazitaxel (as in the TheraP trial), where quantitative PSMA PET may have greater utility in patient selection (7).

In conclusion, the results of this exploratory analysis indicate that the baseline ^68^Ga-PSMA-11 whole-body tumor mean standardized uptake value (SUV_mean_) was the best predictor of ^177^Lu-PSMA-617 efficacy in participants with prostate-specific membrane antigen–positive metastatic castration-resistant prostate cancer in the VISION trial who were selected for the study based on visual read rules. Improvements in radiographic progression-free survival and overall survival were greater among participants with a higher SUV_mean_. Nevertheless, there was evidence for potential clinical benefit with the addition of ^177^Lu-PSMA-617 to the standard of care, regardless of SUV_mean_, in the VISION trial.
